# Fractional-Order Identification of Gyroscope MEMS Noise Under Helium Exposure

**DOI:** 10.3390/s25226954

**Published:** 2025-11-13

**Authors:** Dominik Sierociuk, Michal Macias, Konrad Andrzej Markowski

**Affiliations:** 1 Institute of Control and Industrial Electronics, Warsaw University of Technology, ul. Koszykowa 75, 00-662 Warsaw, Poland; dominik.sierociuk@pw.edu.pl (D.S.); konrad.markowski@pw.edu.pl (K.A.M.); 2 Military Electronic Works JSC, ul. 1 Maja 1, 05-220 Zielonka, Poland

**Keywords:** fractional calculus, fractional Kalman filter, estimation of fractional-order systems, fractional-order noise, mems under helium exposure

## Abstract

This paper tackles the problem of noise analysis and identification in the gyroscope of the LSM06DSO32 inertial navigation sensor based on MEMS technology, under helium exposure. This study focuses on analyzing the bias and variance of the gyroscope noise, as well as identifying its model’s order using fractional-order calculus. The order was estimated using methods based on variance and correlation analysis of data collected from the sensor at various time intervals during helium exposure. This work extends previous research on analyzing and identifying inertial sensor noise under varying temperature conditions. Considering that helium exposure may significantly influence IMU measurements, this study presents a detailed investigation into the evolution of gyroscope noise under prolonged helium exposure, followed by an analysis of the sensor’s behavior after its removal from the helium environment.

## 1. Introduction

Inertial measurement units (IMUs) are ubiquitous components in motion tracking systems, providing real-time data on angular velocity and linear acceleration, which form the basis for estimating spatial position and orientation. However, their precision tends to degrade under adverse environmental conditions.

The reliability of MEMS gyroscopes is closely tied to the integrity of their vacuum packaging, which ensures high-quality-factor (Q-factor) operation and low noise performance. A recurring conclusion across the literature is that helium poses a distinct reliability hazard due to its exceptional diffusivity through common encapsulation materials such as silicon dioxide and polymers [1,2]. Even moderate concentrations of helium can permeate the sensor cavity, increase internal pressure, and significantly degrade resonator performance. This process directly influences bias stability, scale factor accuracy, and noise parameters.

Comparative results of published studies (Table 1) show that the severity of degradation is strongly dependent on the packaging technology. Devices using enclosures sealed with a layer of silicon wafers or epoxy resin exhibit significant vulnerability: polarization deviation changes of several hundred degrees per hour, and in extreme cases exceeding 1000°/h, have been reported after just one week of exposure to moderate helium concentrations [3]. Scale factor errors of several thousand parts per million were also observed, highlighting the serious metrological consequences of helium diffusion [3]. In contrast, gyroscopes enclosed in fully hermetic metal or ceramic housings show negligible changes under the same conditions, as demonstrated by the Epson M-G350 [4].

The mechanism underlying these effects is consistently attributed to helium-induced reductions in Q-factor. Increased cavity pressure diminishes the sharpness of the resonator response, which reduces the signal-to-noise ratio, raises the angular random walk, and accelerates bias drift, as revealed by Allan variance analysis [4,5]. Geiger [6] confirmed this mechanism by showing that MEMS resonators in helium display both a reduced Q-factor and shifts in resonance frequency compared with those in air. These effects directly shorten optimal averaging times and increase bias instability, limiting the suitability of affected devices for high-precision navigation.

More recent work has advanced this understanding. Paper [2] developed a predictive degradation model for wafer-level vacuum-sealed MEMS gyroscopes, linking leak rate and cavity pressure growth with reductions in Q-factor, bias stability, and scale factor accuracy. This study provides the most recent and comprehensive framework for quantifying helium-induced degradation and complements earlier experimental observations.

Mitigation strategies described in the literature include the application of epoxy coatings and the use of barrier layers. While such measures provide partial improvements, none offer complete protection against helium ingress [4]. More effective solutions involve the adoption of alternative barrier materials, such as silicon nitride or atomic-layer-deposited oxides [1,2], or the use of hermetically sealed metal or ceramic packages, which remain the most reliable approach [7,8]. For this reason, helium sensitivity must be treated as a critical selection criterion in applications such as magnetic resonance imaging, semiconductor processing, aerospace and defense, where helium exposure is common.

This work builds on practical experience gained during the development of optoelectronic guidance systems, where MEMS gyroscopes were integrated to monitor angular velocities of the optoelectronic head in both azimuth and elevation axes, forming an essential part of the system’s stabilization and control loop.

The motivation for this investigation emerged from our previous study [9], which analyzed the temperature dependence of gyroscope noise using fractional constant- and variable-order models. Leveraging this background, the current work examines the performance of the same class of sensors under atypical environmental conditions, specifically under helium exposure. The main contribution of this paper is a systematic experimental evaluation of the gyroscope integrated in the LSM6DSO32 IMU sensor manufactured by STMicroelectronics, sourced from Adafruit (New York, NY, USA). This study aims to quantify the effects of helium exposure on the sensor’s noise model, offering new insights into the mechanisms of performance degradation and extending the findings of the earlier temperature-focused research.

Fractional calculus is a mathematical extension of classical calculus that allows differentiation and integration to be performed to arbitrary, non-integer orders [10,11,12]. This approach introduces additional degrees of freedom into mathematical modeling, making it possible to capture complex system dynamics with greater precision and flexibility than traditional integer-order methods. With the growing availability of computational tools and robust numerical methods, fractional-order modeling is becoming a standard approach in areas such as control engineering [13,14] and bioengineering [15,16]. Fractional calculus also encompasses definitions of derivatives and integrals in which the order is time-varying [17,18]. Time-varying fractional-order derivatives can be applied, for example, in the development of control systems [19] or in extensions of unscented Kalman filters based on fractional variable-order definitions [20]. In some cases, time-varying orders can be interpreted in the form of equivalent switching schemes, which also have physical implementations using operational amplifiers [21,22]. These aspects highlight the nature of order variability in selected definitions and support the informed selection of an appropriate formulation when modeling dynamic processes or designing control systems.

This paper is organized as follows. Section 2 begins with an overview of classical integer-order colored noises and then introduces the concept of noise described by fractional variable-order calculus. Section 3 presents the idea of the conducted experiment, including a description of the data acquisition systems and the identification results obtained using variance- and correlation-based methods. This section also includes the analysis of the bias and variance of the investigated noise under helium exposure, with special emphasis on noise order identification using these two approaches. Finally, Section 4 summarizes the identification results and provides concluding remarks.

## 2. Fractional-Order Time-Correlated Noise and Its Identification

Real stochastic processes often exhibit long-range dependencies that cannot be fully described by standard, integer-order noise models. To capture such memory effects, time-correlated noise can be generalized using fractional-order difference operators.

The classical integer-order colored noise is described by

(1) $$v_{k+1}=fv_{k}+\omega_{k},$$

where *f* is a noise parameter and $\omega_{k}$ denotes uncorrelated (typically white) noise.

A more general formulation introduces fractional-order dynamics using a backward difference operator

(2) $$\Delta^{\alpha }v_{k+1}=fv_{k}+\omega_{k},$$

where α∈R is the order of the difference operator $\Delta^{\alpha }$, $v_{k}$ is the time-correlated noise, and $\omega_{k}$ is uncorrelated noise. When α=1, this model reduces itself to the integer-order form. If f=0 and $\omega_{k}$ is Gaussian, Equation (2) corresponds to a realization of a fractional Brownian motion or a fractional Gaussian noise. Time-varying α leads to multifractional noise processes. In multivariable cases, the scalar parameter *f* can be generalized to a diagonal matrix *F* and the scalar order α to a vector, allowing separate fractional behavior for each noise signal.

This fractional-order operator behaves as a derivative when α>0 and as an integrator for α<0 and becomes the identity operator for α=0. Since the infinite sample length of this operator is not practical for computation, a truncated version is used:

(3) Δαxk=∑j=0L(k)(−1)jαjxk−j,

where the αj factor and length of implementation L(k) are defined as

(4) αj=1for j=0α(α−1)…(α−j+1)j!for j>0

and

(5) $$L\left(k\right)=\left\{\begin{array}{cc} k & \mathrm{if}\;k<L,\\ L & \mathrm{if}\;k\geq L. \end{array}\right.$$

The approximation length *L* influences both numerical accuracy and computational cost. Longer memory improves the fidelity of the fractional derivative but increases the complexity of implementation. In this work, the value L=1000 is used for all computations.

Assuming a predefined order α, the noise parameter *f* in Equation (2) can be estimated using the Least Squares (LS) method. Rewriting the system in matrix form yields

(6) D(α,k+1)=fW(k),

where

$$D\left(\alpha ,k+1\right)=\left[\begin{array}{c} \Delta^{\alpha }v_{k+1}\\ \Delta^{\alpha }v_{k}\\ \vdots \\ \Delta^{\alpha }v_{1} \end{array}\right],\;W\left(k\right)=\left[\begin{array}{c} v_{k}\\ v_{k-1}\\ \vdots \\ v_{0} \end{array}\right].$$

The parameter *f* is determined as

(7) f=pinv(W(k))D(α,k+1),

where pinv denotes the pseudoinverse operation. With this, the estimated source noise is given by

(8) $$\omega_{k}^{*}=\Delta^{\alpha }v_{k+1}-fv_{k}.$$

However, in practical applications, the observed signal often includes additional dynamically correlated noise sources. This situation can be modeled as

(9) $$\begin{array}{cc} \Delta^{\alpha }x_{k+1} & =fx_{k}+\omega_{k}, \end{array}$$

(10) $$\begin{array}{cc} v_{k} & =x_{k}+\nu_{k}. \end{array}$$

By applying the fractional difference operator to the observed output $v_{k}$ and substituting the system dynamics, the following expression is obtained:

(11) $$\Delta^{\alpha }v_{k+1}=fv_{k}-f\nu_{k}+\omega_{k}+\Delta^{\alpha }\nu_{k+1}.$$

Consequently, the equation error is as follows:

(12) $$\omega_{k}^{*}=-f\nu_{k}+\omega_{k}+\Delta^{\alpha }\nu_{k+1}.$$

This indicates that the equation error is influenced not only by the intrinsic system noise but also by measurement noise and its fractional difference. The presence of these multiple noise sources complicates the accurate identification of the fractional-order model.

To address this challenge, two methods are proposed for estimating the order. Both approaches rely on analyzing the properties of the estimated source noise, which reflects the mismatch between the observed signal and the assumed noise model.

The first method focuses on the variance of the estimated source noise. It assumes that the optimal value of order results in the smallest noise variance. A minimal variance indicates that the model successfully captures the underlying dynamics.

The second method is based on minimizing a quality index proposed in [23], which measures the time correlation of the estimated source noise. The quality index is defined as the sum of absolute values of the autocorrelation function:

(13) $$J=\sum_{m=0}^{k}\left|R_{\omega^{*}}\left(m\right)\right|,$$

where

(14) $$R_{\omega^{*}}\left(m\right)=E\left\{\omega_{k+m}^{*}\omega_{k}^{*}\right\}=E\left\{\omega_{k}^{*}\omega_{k-m}^{*}\right\},$$

and *m* is the shift between samples.

The variance-based and correlation-based methods make it possible to estimate the fractional order by selecting the model that best matches the observed noise characteristics.

## 3. Experimental Results

The main idea considered in this paper is to analyze the influence of exposing the gyro sensor to a helium environment on the gyro noise parameters. We can expect the reaction time for helium exposure to be rather long (in hours) because it must penetrate the sensor. For obtaining experimental data, the LSM6DSO32 gyro sensor was used. The maximum frequency that allows reading of data is 6660 Hz. For such a sampling frequency, it is possible to obtain data without additional digital signal processing (by discrete filters). Given the high value of sampling time and long collection time, it was necessary to develop a specialized method of data acquisition. To control these processes, the Arduino Portenta H7 board, paired with the Arduino Portenta Breakout, was used (Arduino, Monza, Italy). This board is built based on the STM32H747XI dual Cortex^®^-M7 + M4 32-bit low-power Arm^®^ MCU, which has two cores: Cortex^®^ M7 running at 480 MHz and Cortex^®^ M4 running at 240 MHz. One core is dedicated to communication with the sensor, and the second core is dedicated to communication with the SD card for saving the obtained data. Due to restrictions implemented in Arduino Portenta FAT32, the maximum amount of saved data in one acquisition round was 4 GB, which allows for collecting data for approximately 12 h of continuous acquisition. That is why the collected data will be divided into separate sequences.

### 3.1. Experimental Setup

**Figure 1:**
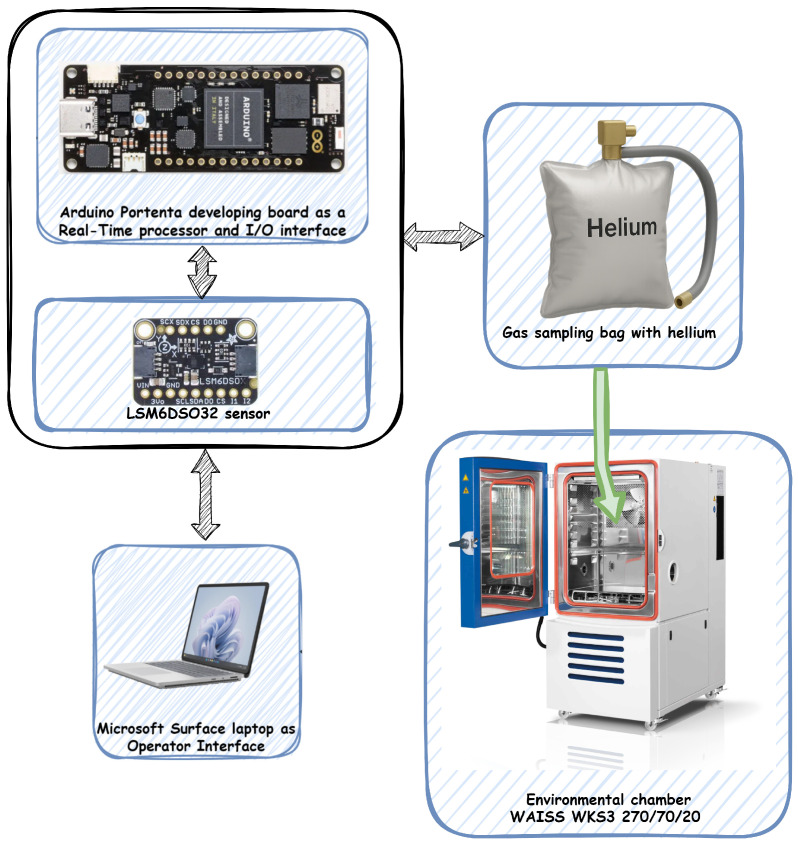
Environment setup.

The configuration used in this study included several integral components (Figure 1): a Microsoft Surface laptop functioning as the Operator Interface; an Arduino Portenta development board equipped with a dual-core STMicroelectronics STM32H747 processor (STMicroelectronics NV, Geneva, Switzerland) for real-time processing and I/O operations; a gas sampling bag; and an environmental chamber manufactured by Weiss Technik (model WAISS WKS3 270/70/20, Weiss Technik GmbH, Regierungsbezirk Gießen, Germany).

The LSM6DSO32 IMU sensor, as a tested component, was first placed inside a gas sampling bag filled with technical helium. The gas sampling bag is made of low-permeability materials, such as aluminium foils with barrier coatings or fluoropolymers (FEP, Tedlar, Adtech Polymer Engineering, Stroud, UK). Thanks to their multilayer structure, they are resistant to gas permeation as well as mechanical damage. The helium-filled bag containing the sensor was then placed inside the environmental chamber. This created a helium-rich environment immediately surrounding the IMU, allowing us to assess its performance under these specific conditions. Throughout the experiment, the chamber maintained a constant temperature of 22 °C to ensure thermal stability, and the atmospheric pressure was set to 1012 hPa. To avoid introducing additional noise or disturbances into the system that could compromise the data collection, helium was only added to the gas bag prior to the start of each measurement sequence. Consequently, no helium was introduced during the measurement process.

### 3.2. Measurement Methodology

The measurement setup was based on the STMicroelectronics LSM6DSO32 inertial measurement unit (IMU) and the Arduino Portenta development board. The LSM6DSO32 is a MEMS-based IMU that integrates a three-axis accelerometer and a three-axis gyroscope. It supports I2C and SPI communication interfaces and offers a maximum sampling frequency of 6.6 kHz. The accelerometer range is configurable up to ±32 g, providing a digital resolution of approximately 0.976 mg at ±32 g (16-bit output), while the gyroscope range reaches ±2000 dps, with a digital resolution of about 0.061 dps (16-bit output).

Data acquisition and processing were handled by the Arduino Portenta board, which features a dual-core STM32H747 processor, 8 MB of SDRAM, three 16-bit A/D converters, and two 12-bit D/A converters—providing adequate resources for efficient, real-time operation throughout the experiment.

Due to the limitation of approximately 12 h available for continuous data collection, the measurement process was divided into a series of acquisition sequences, as presented in Table 2.

As can be seen in Table 2, we have acquired one reference dataset before helium exposure, five data sequences during helium exposure, and four sequences after helium exposure (one sequence after a long time to study the long-term effect of helium exposure).

The data collected during acquisition were as follows: gyro measurements from one axis, the time of obtaining the data, and a sample loss indication (to ensure that all data were collected properly). In one sequence (approximately 12 h of measurements), 187,159,392 samples were collected. In processing, the data were divided into 10,000 samples of fixed-length chunks (yielding 18,715 chunks) to better analyze changes in mean and variance values. To begin with, the offset value of the measured signal will be taken into consideration.

### 3.3. Gyro Bias Analysis

The bias will be analyzed as the mean value of the samples in each chunk separately (for 10,000 samples).

**Figure 2:**
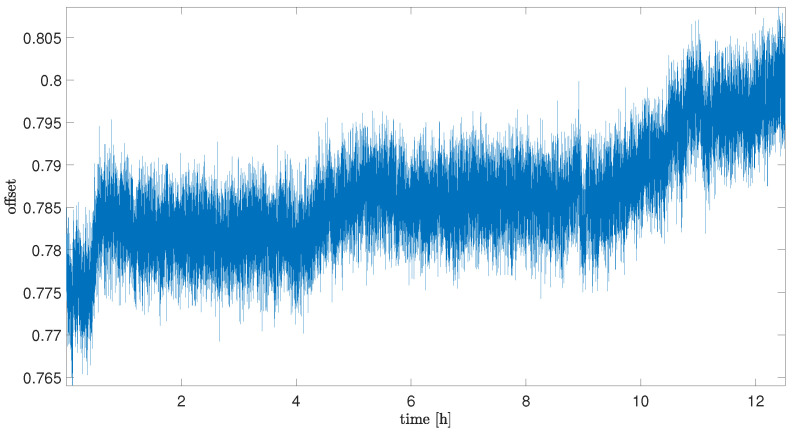
Offset of the sensor for the measurement before helium exposure (sequence ref).

**Figure 3:**
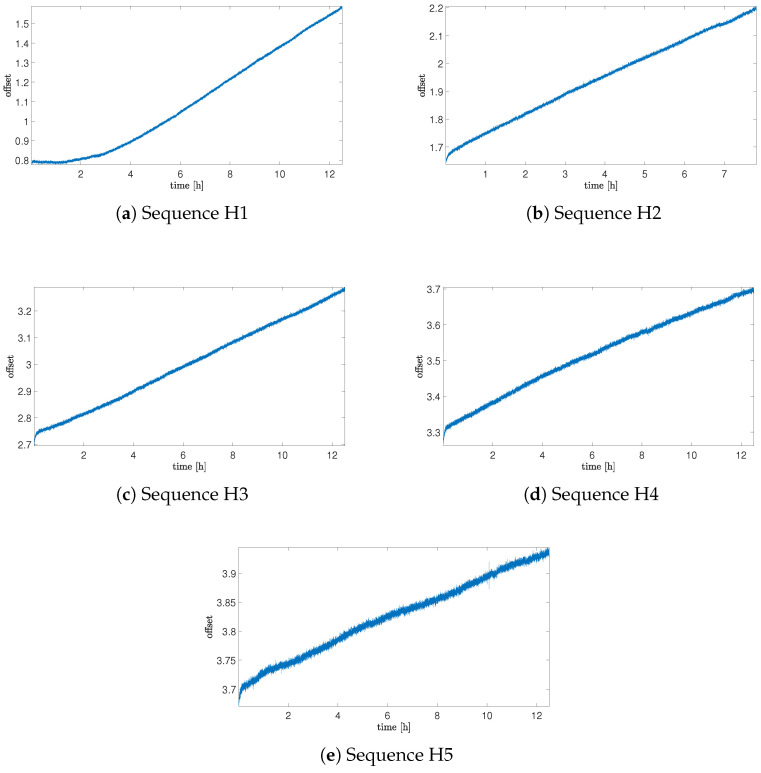
Offset of the sensor for sequences of measurements with helium exposure.

**Figure 4:**
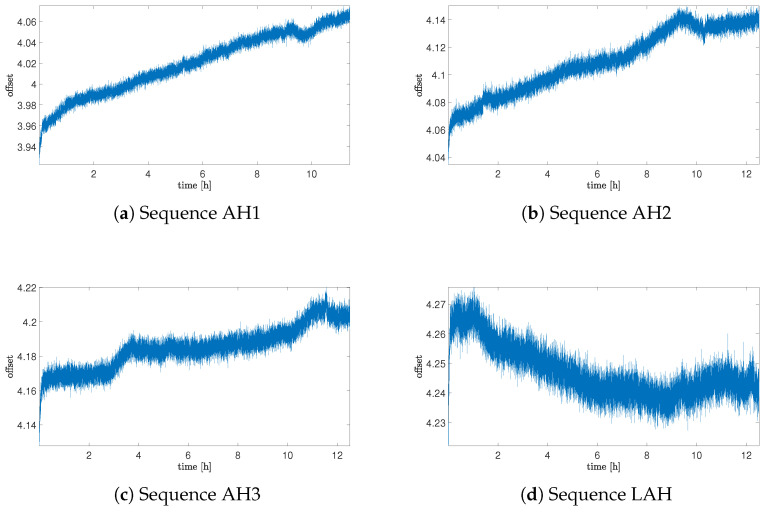
Offset of the sensor for sequences of measurements after helium exposure.

Results of offset analysis for all cases of sequences are presented in Figure 2, Figure 3 and Figure 4.

Figure 3 presents results of changes in noise offset during the time of sensor exposure to the helium gas. As can be noticed, there is a significant influence on offset drift values (which was expected when taking into account the literature presented in Table 1). After 1.5 h of exposure, a high increase in the sensor noise bias can be noticed. In comparison to the reference value, the offset drift increases about 20 times more, which clearly emphasises the strong influence of the helium exposure on this MEMS sensor. As can also be noticed, this influence, after increasing at the beginning, decreases with the time of the exposure. The average value of offset drift for Sequence 5 (the last collected sequence during helium exposure) is about three times less than for the first collected sequence, but still about six times more than for the reference sequence.

After observing the influence on the helium exposure stage, it was worth analyzing the behavior of the noise offset in the situation where the sensor is exposed to air again (after stopping exposure to helium). The main issue was to recognize if the observed effect would be permanent.

In Figure 4, the offset data for the situation after stopping the helium exposure are presented. As can be noticed, the offset drift decreases with time, but obtaining a drift comparable to this from the reference sequence took much more time than for exposure—it was observed in the sequence after 30 days of exposure. It is essential to note that the value of the offset itself does not return to the initial range, but remains permanently within the range of the offset observed at the end of the helium exposure.

Offset drift data are summarized in Table 3. It is important to notice that for Sequence H1, the average value of the drift was obtained for the whole time range of acquisition and also included the beginning part when the influence of the helium exposure was not observed. The average drift value, excluding the initial delay, was 0.0774 and was higher than for Sequence H2.

### 3.4. Variance Analisys

Previous results showed a strong influence of helium exposure on the offset value; in this section, analysis of the measurement noise variances will be taken into consideration.

**Figure 5:**
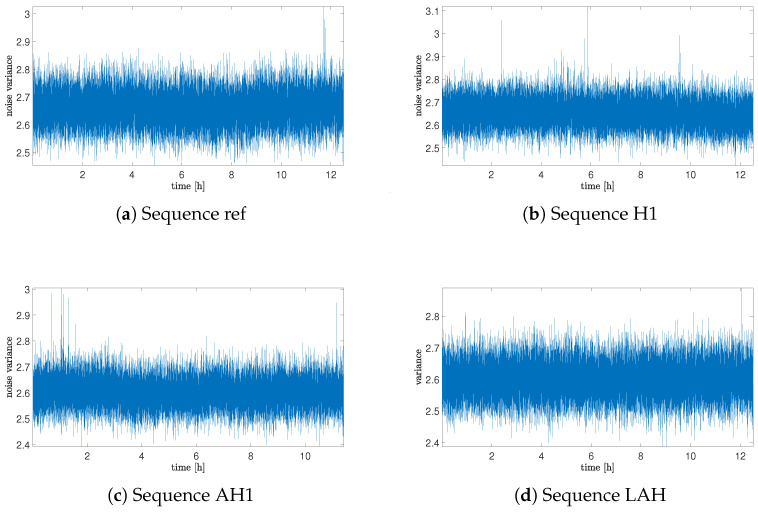
Variance plots of the sensor for chosen sequences of measurements.

Figure 5 presents plots of variances obtained for 10,000 sample chunks separately. Plots are presented only for chosen sequences due to space limitations. As can be noticed from these plots, the variance does not seem to have any evident or meaningful rule of variability.

More precisely, results are composed in Table 4, with the mean value of variance for the whole sequence and variance of variances obtained for each chunk separately. The data presented in the table were obtained for all sequences, and as can be seen, values of variance and variance of variances differ from each other only marginally. This suggests that the helium exposure does not have an effect on the noise type; however, additionally, tests based on the identification of fractional-order noise will be needed.

### 3.5. Order Identification Based on Variance and Correlation Methods

In this section, the results of the analysis based on the fractional-order noise identification algorithm will be presented and verified. In contradiction to previous results of variance, these results will be focused on the source noise—the noise obtained as an error of the LS identification process (see Equation (12)). Two methods for analysis will be used: variance and correlation analysis methods. The variance method assumes that the identified order occurs for a minimum of source noise variance. Otherwise, for correlation-based analysis, the identified orders occur for a minimum correlation of the source noise.

**Figure 6:**
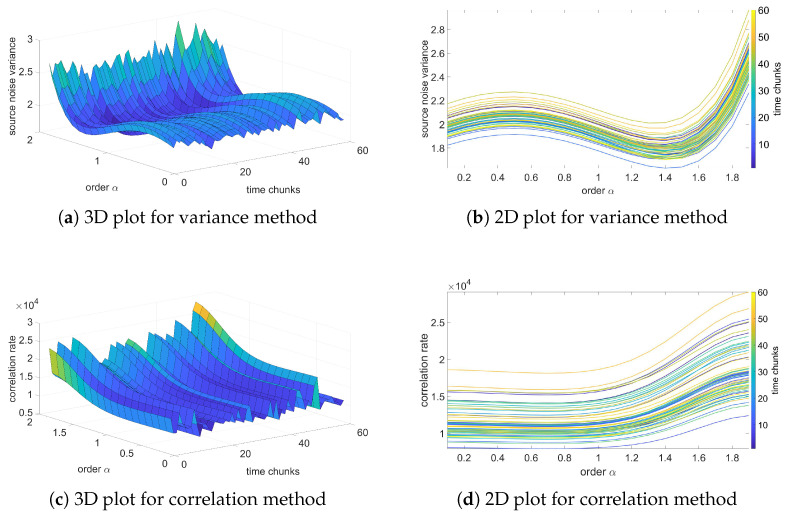
Variance and correlation method plots for the reference sequence.

**Figure 7:**
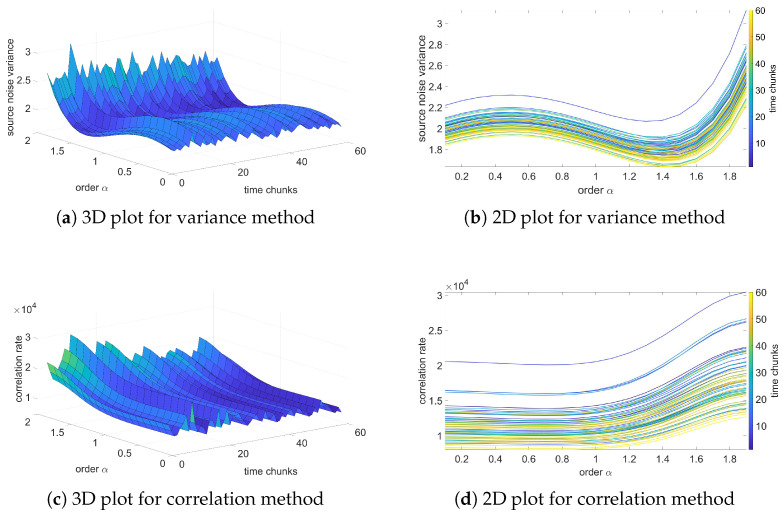
Variance and correlation method plots for Sequence H1.

**Figure 8:**
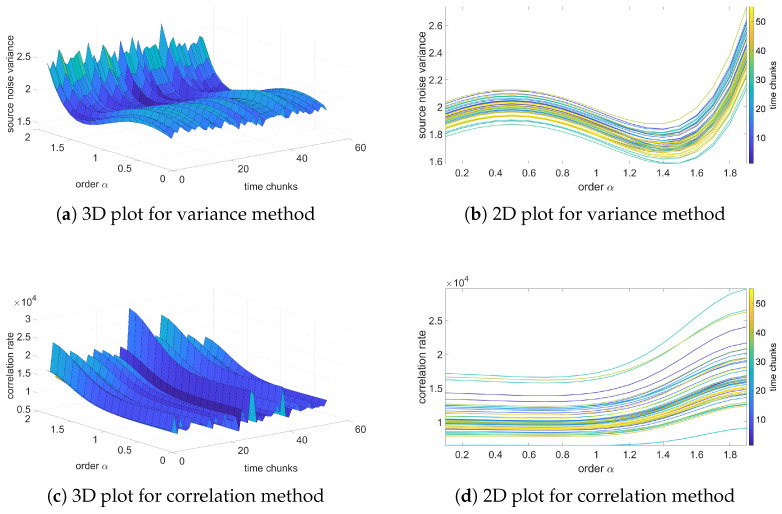
Variance and correlation method plots for Sequence AH1.

**Figure 9:**
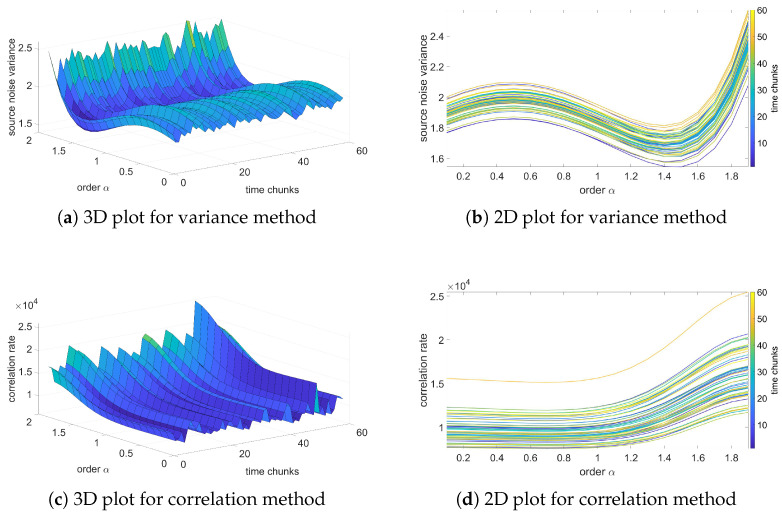
Variance and correlation method plots for Sequence LAH.

Figure 6, Figure 7, Figure 8 and Figure 9 present the results of the order analysis based on the variance and correlation methods for the chosen sequences. As can be noticed, the obtained plots do not present a clear dependency, as was the case for a temperature relation in [9]; the surface of the 3D plot has a rather noisy character. However, the minimum of each plot for particular chunks is nearly at the same point, which led to the conclusion that the order of the noise does not depend on helium exposure.

More detailed results are summarized in Table 5 and Table 6, and we can clearly observe that the estimated order for the variance method is nearly the same for all sequences, also with a very low value of variance. For the correlation method, the changes are marginally larger and have higher variance, but still remain at negligible values. These results confirm observations from variance analysis from the previous section that the character of noise does not depend on exposure to the helium; the influence is only in the offset of the noise.

The estimated orders obtained from these two methods differ from each other; the variance method yields an order of approximately 1, while the correlation method yields an order of about 1.4. A similar difference was also observed between these two methods in the case of temperature noise, as seen in [23]. In [9], an alternative method based on estimation analysis is presented, showing that the correlation method yields more accurate results for the estimation process. However, this method is more computationally intensive. For observing order-changing dependency, both methods used in this paper provide sufficient results.

## 4. Conclusions

This paper was devoted to analyzing the influence of exposing the MEMS sensor (specifically the gyroscope from the particular example of the LSM6DSO32 sensor) to helium gas. The analysis focused on gyroscope noise parameters, including offset (bias), variance, and the type of dynamical correlation in the noise—specifically, the fractional order of the noise. The model fractional-order noise was identified using two approaches: variance analysis and a selected correlation indicator applied to data collected at different time intervals.

Experimental data were collected in 10 sequences in various situations: 1—reference sequence—before helium exposure, 5 sequences during helium exposure, and 4 sequences after stopping exposure.

A detailed examination of the noise measurements leads to the observation that there is a strong influence on the offset when the sensor is exposed to the helium-filled environment. Under these conditions, the offset increases over time. Such behavior is similar to that presented in the literature collected in Table 1. In this paper, we use less pressure (atmospheric) than in other articles and analyze the fractional noise order. This is because what is also interesting—in opposition to the offset—is that the noise variance shows only marginal and negligible changes under helium exposure. This suggests that exposing the sensor to the helium environment does not significantly affect this parameter.

Further analysis of the sensor’s dynamic model, based on fractional-order noise methods, shows that across different data segments collected at specific intervals, the model order remains largely consistent, with only small deviations. In the case of model order identification using the variance-based method, a value of approximately 1.4 was obtained, while the correlation-based approach yielded a value of 1. Despite this difference between the two methods, the noise model maintains a constant order. This insight can be used when modeling noise in scenarios where computational complexity needs to be minimized. Applying a fractional but constant-order model is less computationally demanding than using a model with a varying order, which requires more resources and a longer processing time.

The offset drift after exposing the sensor to the helium with some delay at the beginning of the exposure starts to increase to a value about 20 times higher than for the reference sequence, and the last measurement during exposure shows that the value of the drift is about six times more than the reference value. After stopping, the exposure value of the drift decreases with time, but much more slowly than for the exposure stage. A range close to reference sequence values was measured after 30 days of exposure. Most notably, the drift comes back to the range of the reference sequence, but the value of the offset itself does not—the offset seems to be changed permanently. During the whole experiment, the offset increased from a value of 0.775 to 4.242.

**Figure 10:**
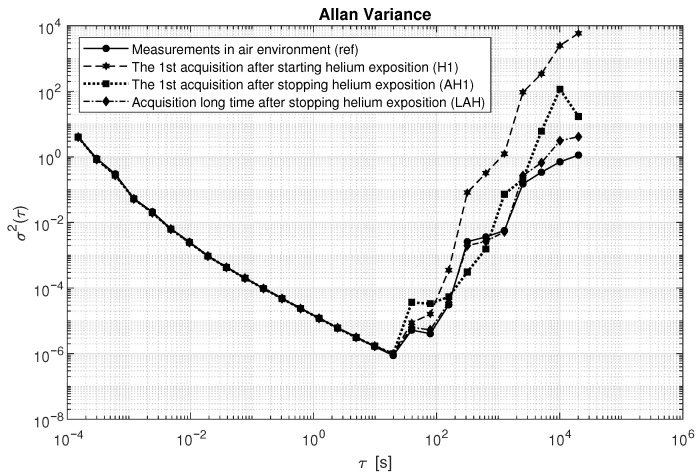
Comparison of Allan variance for different data sequences exposed to helium.

To complement the noise analysis, Figure 10 presents a comparison of the Allan variance for the considered datasets. It can be observed that the estimated values coincide up to the point where the minimum is reached. Differences start to appear at averaging times of around 20 s, where the dataset recorded after starting helium exposure shows the steepest increase and reaches the highest values. This behavior is attributed to the growth in long-term drift resulting from helium influence. Sensor errors clearly increase for longer averaging times, particularly for the H1 sequence, which corresponds to the dataset collected during helium exposure. The Allan variance characteristics for the dataset recorded a long time after stopping helium exposure are the most similar to the reference data.

Considering that the helium concentration in the air is very small (approximately 5.2 ppm), it can affect the sensor very slowly, providing an indicative insight into the changes that may occur during the sensor’s aging process. This can give very important information in the maintenance management process. However, in the conducted study, the main focus was on the nature of the order of the model and its constancy, while other aspects of parametric noise modeling will be considered as future research directions.

## Figures and Tables

**Table 1:** Comparative findings on helium effects in MEMS gyroscopes.

Source	Device/Packaging	Test Conditions	Bias Drift	Scale Factor Error	Q-Factor/Notes
Hilgemann et al. (2016) [4]	Murata SCR1100-D04, ST L3G4200D, Epson M-G350	Controlled He chamber	Significant for Murata and ST; negligible for Epson	Degradation in Murata and ST; stable in Epson	Allan variance showed increased noise; hermetic Epson resistant; epoxy coating partial improvement
Mathisen (2022) [3]	STIM gyros (wafer-level)	7 days, 800 kPa, 40 ppm He	Median +157°/h; worst +1277°/h	Avg. 6919 ppm	Severe degradation; recommends avoiding helium-rich environments
Sparks et al. (2013) [5]	Vacuum-packaged MEMS sensors	Room-temperature He exposure	Output drift (qualitative)	—	Reduced Q-factor; effects partly reversible
Kim et al. (2009) [1]	MEMS encapsulated with polysilicon	Diffusion tests	—	—	He and H_2_ fastest-diffusing gases; SiO_2_ main path
Xu et al. (2023) [2]	Wafer-level MEMS gyros	Simulation + experiments	Bias drift grows with pressure rise	Scale factor error increases with leakage	Q-factor decreases as cavity pressure rises; predictive degradation model
Geiger (2012) [6]	MEMS resonator	Air vs. He at varying pressures	—	—	Q-factor reduced in He; resonance frequency shift
Choa et al. (2005) [7]	MEMS gyros, anodic bonding	Long-term reliability tests	—	—	Q-factor decreases with leakage; confirms packaging leakage critical
Passaro et al. (2017) [8]	MEMS gyros (review)	Literature survey	Notes helium as bias drift source	—	Situates helium sensitivity within broader gyro reliability issues

**Table 2:** Descriptions of sequences with acquired data.

Sequence Name	Sequence Description
sequence ref	measurements in air environment before helium exposure (reference measurements)
sequence H1	the 1st acquisition after starting helium exposure
sequence H2	the 2nd acquisition during helium exposure
sequence H3	the 3rd acquisition during helium exposure
sequence H4	the 4th acquisition during helium exposure
sequence H5	the 5th acquisition during helium exposure
sequence AH1	the 1st acquisition after stopping helium exposure (air environment)
sequence AH2	the 2nd acquisition after stopping helium exposure (air environment)
sequence AH3	the 3rd acquisition after stopping helium exposure (air environment)
sequence LAH	acquisition a long time (30 days) after stopping helium exposure (air environment)

**Table 3:** Results of offset for different stages of measurement process.

Sequence Number	Offset Drift [deg/h]
sequence ref	0.003565
sequence H1	0.064995
sequence H2	0.072974
sequence H3	0.047426
sequence H4	0.035417
sequence H5	0.021916
sequence AH1	0.013351
sequence AH2	0.009225
sequence AH3	0.007388
sequence LAH	0.004292

**Table 4:** Results of variances for different stages of the measurement process.

Sequence Number	Mean Value of Noise Variance	Variance of Noise Variance
sequence ref	0.003414	2.659456
sequence H1	0.003455	2.651910
sequence H2	0.003211	2.633650
sequence H3	0.003042	2.608097
sequence H4	0.003413	2.611220
sequence H5	0.003002	2.604006
sequence AH1	0.003068	2.601916
sequence AH2	0.003071	2.602575
sequence AH3	0.002973	2.599731
sequence LAH	0.002917	2.599508

**Table 5:** Results of order estimation based on the variance method for different sequences.

Sequence Number	Estimated Order	Variance of Estimated Order
sequence ref	1.398333	0.000167
sequence H1	1.400000	0.000339
sequence H2	1.400000	0.000000
sequence H3	1.400000	0.000339
sequence H4	1.396667	0.000667
sequence H5	1.400000	0.000000
sequence AH1	1.401818	0.000182
sequence AH2	1.405000	0.000483
sequence AH3	1.400000	0.000000
sequence LAH	1.403333	0.000328

**Table 6:** Results of order estimation based on the correlation method for different sequences.

Sequence Number	Estimated Order	Variance of Estimated Order
sequence ref	1.010000	0.158881
sequence H1	1.066667	0.152090
sequence H2	0.989474	0.146373
sequence H3	1.073333	0.136565
sequence H4	1.021667	0.150201
sequence H5	1.091667	0.175692
sequence AH1	1.050909	0.193657
sequence AH2	0.986667	0.146599
sequence AH3	1.036667	0.134565
sequence LAH	1.115000	0.156890

## Data Availability

The original contributions presented in this study are included in the article. Further inquiries can be directed to the corresponding author.
